# Binder-Free Fe_2_O_3_/MWCNT/Al Electrodes for Supercapacitors

**DOI:** 10.3390/nano15161222

**Published:** 2025-08-10

**Authors:** Alena A. Mitina, Evgene E. Yakimov, Maxim A. Knyazev, Victor I. Korotitsky, Arkady N. Redkin

**Affiliations:** Institute of Microelectronics Technology and High-Purity Materials, Russian Academy of Science (IMT RAS), Moscow District, 6 Academian Ossipyan Str., 142432 Chernogolovka, Russia

**Keywords:** carbon nanotubes, hematite, binder-free electrodes, supercapacitor

## Abstract

This work presents a method for preparing an Fe_2_O_3_/MWCNT/Al composite electrode without the use of a binder. Synthesizing the composite material directly on conductive substrates allows one to obtain ready-made supercapacitor electrodes characterized by high values of specific capacity, as well as resistance to numerous charge/discharge cycles. Using an array of multi-walled carbon nanotubes (MWCNTs) as a conductive base for the synthesis of iron oxide allows for the production of a composite material that combines the positive properties of both materials. The Fe_2_O_3_/MWCNT/Al composite was formed using electrochemical oxidation of the MWCNT/Al material in a mixture of 0.1 M aqueous solution of Fe(NH_4_)_2_(SO_4_)_2_ (iron ammonium sulfate) and 0.08 M CH_3_COONa (sodium acetate) in a 1:1 ratio. The proposed approaches to fabricating composite electrodes provide excellent performance characteristics, namely high cyclic stability and fast response time. For the first time, an Fe_2_O_3_/MWCNT/Al composite was obtained using electrochemical oxidation of Fe^2+^ on the surface of MWCNTs grown directly on aluminum foil. The specific capacitance of the obtained composite material reaches 175 F/g at a scanning rate of 100 mV/s. The capacity loss during cyclic measurements does not exceed 25% after 10,000 charge/discharge cycles.

## 1. Introduction

Creating reliable electrochemical energy storage devices is a primary task for the storage, conversion, and further use of renewable (clean) energy [1,2,3]. There are two main types of energy storage and accumulation devices: supercapacitors and lithium-ion batteries [4,5,6]. Supercapacitors (SCs) are electrochemical energy sources characterized by high power, long life, and high stability over multiple charge/discharge cycles, as well as safety and environmental friendliness [7,8,9]. The performance of supercapacitors directly depends on the active material of the electrode [10,11]. Much work has been performed using transition metal oxides as active materials of electrodes [12,13,14]. The redox behavior of these oxides is related to the multivalent nature of transition metals. Promising materials include iron-based materials such as hematite (Fe_2_O_3_) [15,16,17], magnetite (Fe_3_O_4_) [18], FeOOH [19], FeO_x_ [20], and CoFe_2_O_4_ [21]. They have attracted attention as promising materials for SC electrodes due to their high theoretical capacitance, wide negative potential range (−1.2–0 V), abundance in nature, low cost, and non-toxicity [22,23,24]. Despite their high values of specific capacitance, most transition metal oxides have low conductivity, poor stability, a low charging rate, and a short life during the charge/discharge process [25,26]. One way to improve the conductivity of iron-based materials is to combine them with conductive carbon materials [27,28,29]. Due to the synergistic effect of the high capacitance of iron oxides and the high conductivity of carbon materials, composite materials demonstrate better electrochemical characteristics than the original pseudocapacitive materials [30,31,32]. Carbon nanotubes are characterized by high conductivity, a large specific surface area, mesoporosity, environmental friendliness, and chemical stability. These properties make them a promising material for supercapacitor electrodes [33,34].

Conventional electrode manufacturing processes use binders, which usually have high resistivity and low electrochemical activity. Their use degrades the electrochemical performance of the electrode by increasing the internal resistance [35]. In addition, conventional binders are often based on organic solvents, which can affect the environment. Therefore, the study of binder-free electrode preparation methods has become an important area of research to improve the performance of supercapacitors. The preparation of binder-free electrodes increases the amount of active material in the electrodes and also significantly increases the conductivity of the electrodes. One way to prepare binder-free electrodes is to grow electrochemically active materials on conductive substrates [36].

Synthesis of MWCNTs on metal substrates can improve electrochemical properties due to a decrease in the contact resistance between the active electrode material and the current collector [37,38]. Aluminum is a promising material for supercapacitor electrodes due to its high electrical conductivity and plasticity [39]. Electrodes made of aluminum foil coated with an MWCNT layer are suitable for use in flexible supercapacitors [40]. The aluminum foil acts as a conductive substrate for the synthesis of the MWCNT array and as a current collector for the finished electrode. In turn, the MWCNT array acts as a conductive base for the formation of an iron oxide layer and as the active material in the supercapacitor electrode. Composite electrodes based on carbon nanotubes with transition metal oxides combine physical and chemical mechanisms of charge accumulation on a single electrode. Nanotube-based materials promote charge generation through the formation of an electric double-layer capacitor (EDLC) and also provide a large specific surface area [41], which in turn increases the contact area between the electrolyte and the pseudocapacitive materials applied to the MWCNTs. In this case, pseudocapacitive materials increase the specific capacitance values due to fast redox reactions [42,43]. There are various technologies for the production of composite materials. Methods such as hydrothermal deposition [44], electrochemical deposition [45,46,47,48,49], and electrospinning [50] are used to produce an electrode without a binder.

In this work, the possibility of producing a composite electrode based on multi-walled carbon nanotubes and iron oxide without the use of binders was investigated. Thin films of Fe_2_O_3_ were deposited on the surface of carbon nanotube arrays grown on aluminum foil with electrochemical deposition. The optimal conditions for preliminary electrochemical oxidation of the carbon nanotubes were selected: 10 min in 0.005 M aqueous Na_2_SO_4_ solution at an oxidation voltage of 4 V. This allowed us to obtain a composite electrode with high resistance to numerous charge/discharge cycles (10,000). The dependence of the specific capacity and stability of the electrode material on the annealing temperature was studied. The electrochemical properties of the composite were studied as a function of the parameters of iron hydroxide film formation on the surface of the carbon nanotube array. The optimal conditions for composite formation were selected, which led to a fivefold increase in specific capacitance: electrochemical oxidation of MWCNT/Al samples in an aqueous solution (Fe(NH_4_)_2_(SO_4_)_2_ 0.1 M and CH_3_COONa 0.08 M) at a voltage sweep rate of 2 mV/s, followed by annealing at 300 °C. All samples were characterized by scanning electron microscopy (SEM), Raman spectroscopy (RS), X-ray photoelectron spectroscopy (XPS), cyclic voltammetry (CV), galvanostatic charge/discharge (GCD), and electrochemical impedance spectroscopy (EIS) methods.

## 2. Materials and Methods

### 2.1. Synthesis of MWCNTs on Aluminum Foil

The synthesis of carbon nanotubes was carried out using a previously developed method [51]. A CVD synthesis setup was used for deposition. MWCNTs were deposited at atmospheric pressure onto aluminum foil substrates (99.0%, Russia) coated with a catalyst layer. The deposition temperature was 600 °C, and the synthesis duration was 1 h. Ethanol consumption was 6–7 mL/h (96 wt.%, Russia).

### 2.2. Synthesis of Fe_2_O_3_/MWCNT/Al Composite Material

Pre-oxidized MWCNT/Al samples were used to prepare the Fe_2_O_3_/MWCNT/Al composite material. Electrochemical oxidation of the MWCNT/Al material was carried out in a two-electrode cell. A platinum wire served as a counter electrode. A strip of aluminum foil coated with an MWCNT layer acted as an anode during the oxidation process. A 0.005 M Na_2_SO_4_ (high purity grade, Russia) solution was used as an electrolyte. The oxidation time was 10 min at a voltage of 4 V [52]. A three-electrode electrochemical cell was used to form the Fe_2_O_3_/MWCNT/Al composite material. The MWCNT/Al sample served as the working electrode. A saturated calomel electrode was used as a reference electrode, and a pure platinum wire was used as a counter electrode.

The process was carried out using a P-40X potentiostat (Electrochemical Instruments, Chernogolovka, Russia). The electrolyte was a mixture of 0.1 M aqueous solution of Fe(NH_4_)_2_(SO_4_)_2_ (iron ammonium sulfate (special purity grade, Russia)) and 0.08 M CH_3_COONa (sodium acetate (special purity grade, Russia)) in a 1:1 ratio. The voltage range was from −10 to 700 mV, and the voltage sweep rate was from 2 to 100 mV/s. After the formation of the iron hydroxide layer, the samples were washed in distilled water and dried in air for 24 h. The resulting material was annealed in air at temperatures of 200, 300, and 400 °C. The heating rate for the formation of the composite material was 15 °C/min. For further studies, samples prepared as follows were selected:

(a) Synthesis of MWCNTs on the surface of aluminum foil, electrochemical oxidation of MWCNTs;
(b) Synthesis of MWCNTs on the surface of aluminum foil, electrochemical oxidation of MWCNTs, formation of an iron hydroxide layer on the surface of MWCNTs at a voltage sweep rate of 2 mV/s, calcinations at a temperature of 200 °C;
(c) Synthesis of MWCNTs on the surface of aluminum foil, electrochemical oxidation of MWCNTs, formation of an iron hydroxide layer on the surface of MWCNTs at a voltage sweep rate of 2 mV/s, calcinations at a temperature of 300 °C;
(d) Synthesis of MWCNTs on the surface of aluminum foil, electrochemical oxidation of MWCNTs, formation of an iron hydroxide layer on the surface of MWCNTs at a voltage sweep rate of 2 mV/s, calcinations at a temperature of 400 °C.

### 2.3. Structural Characterization

The Raman spectra of the obtained samples were studied in the range of 150–2000 cm^−1^ with a spectral resolution of 9–15 cm^−1^ using a Bruker Senterra micro-Raman system coupled to an OLYMPUS BX51 optical microscope. The spectra were recorded under excitation by a solid-state laser at a wavelength of 532 nm. The laser operating power was 5 mW. X-ray photoelectron spectra of the sample were obtained on a SPECS electron spectrometer with a FOIBOS-150 energy analyzer (SPECS GmbH, Berlin, Germany) using non-monochromatic Al Kα radiation (1486.6 eV). The spectra were measured at an anode voltage of 12.5 kV and an emission current of 19.0 mA on the XR50 X-ray source. The spectra obtained were processed using CasaXPS software (version 2.3.25PR1.0). To compare and study the morphology of the samples obtained, an AURIGA CrossBeam microscope (Carl Zeiss NTS, Germany) was used in scanning mode with an acceleration voltage of 5 kV. An attachment to the JEOL 6490 INCA Oxford Instruments electron microscope was used to study the elemental composition of the samples. The electron accelerating voltage was 15 kV, and the current was ~1 nA.

### 2.4. Electrochemical Measurements

Electrochemical tests on the original and modified samples (cyclic voltammetry, impedance spectroscopy) were carried out in a three-electrode cell. A saturated calomel electrode was used as the reference electrode. A platinum wire was used as the counter electrode. The working electrode was a strip of aluminum foil (0.75 × 2 cm^2^) coated on both sides with a composite material. A 0.5 M aqueous solution of Na_2_SO_4_ was used as the electrolyte. Electrochemical impedance measurements were carried out in the presence of a 0.5 M aqueous solution of Na_2_SO_4_ at a constant potential of 0 V, superimposed on an alternating potential with an amplitude of 20 mV in the frequency range from 50 kHz to 100 mHz. Electrochemical measurements were performed using a P-40X potentiostat equipped with an FRA-24M frequency analyzer module (Electrochemical Instruments, Chernogolovka, Russia).

## 3. Results and Discussion

### 3.1. Comparison of Particle Size and Morphology Based on the SEM Images and Discussion of the Influence of Temperature

An array of carbon nanotubes grown directly on aluminum foil can serve as a base for obtaining Fe_2_O_3_/MWCNT/Al composites with high specific capacitance values due to the pseudocapacitive properties of iron oxide. An analysis of the literature shows that the functionalization of the carbon material plays an important role in the formation of an iron oxide layer on the surface of a carbon material [53]. The technique of electrochemical oxidation in a low Na_2_SO_4_ solution allows for the optimal conditions for processing the MWCNT/Al material to be selected. It is important to select conditions for pre-oxidation of the sample that not only ensure further formation of the composite but also do not lead to the destruction of the MWCNT layer. During the experiments, it was found that the optimal processing conditions are achieved with electrochemical oxidation of MWCNT/Al samples for 10 min in 0.005 M aqueous Na_2_SO_4_ solution at an oxidation voltage of 4 V. Fe(NH_4_)_2_(SO_4_)_2_ was used as the source of iron in the formation of the composite. CH_3_COONa was used as a reducing agent to prevent destruction of the MWCNT layer. An iron hydroxide layer was formed on the MWCNT surface [54]. The resulting material was annealed in air at 200, 300, and 400 °C to form the Fe_2_O_3_/MWCNT/Al composite material.

To select the optimal conditions for the preparation of the composite, preliminary tests were carried out at different voltage sweep rates. Energy-dispersive X-ray spectroscopy (EDX) of the surface layer of all processed samples showed the presence of carbon, oxygen, and iron as the main components of the active layer. Data on their concentrations are given in Table 1.

**Figure 1:**
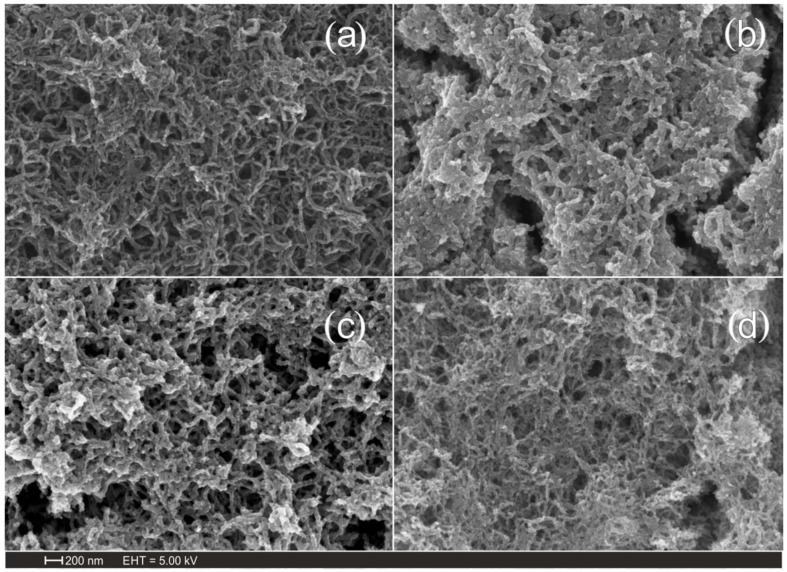
SEM images of (**a**) MWCNT/Al, (**b**) 200 °C—Fe_2_O_3_/MWCNT/Al, (**c**) 300 °C—Fe_2_O_3_/MWCNT/Al, and (**d**) 400 °C—Fe_2_O_3_/MWCNT/Al.

The results obtained show that, at a voltage sweep rate of 2 mV/s, the composite material contained the greatest amount of not only iron but also oxygen. The samples obtained at a voltage sweep rate of 2 mV/s were selected for further investigation. To convert the iron hydroxide layer to oxide, the material was annealed at 200, 300, and 400 °C. The morphology of the composite material was studied using scanning electron microscopy. Figure 1 shows the scanning electron microscopy images of carbon nanomaterials (Figure 1a) and Fe_2_O_3_/MWCNT/Al composites (Figure 1b–d). The studies were carried out on samples obtained at annealing temperatures of 200 (Figure 1b), 300 (Figure 1c), and 400 °C (Figure 1d). In all cases, we observed intertwined MWCNTs, and in the case of the composite material, the surface of the carbon nanotubes was covered with a layer of iron oxide.

**Figure 2:**
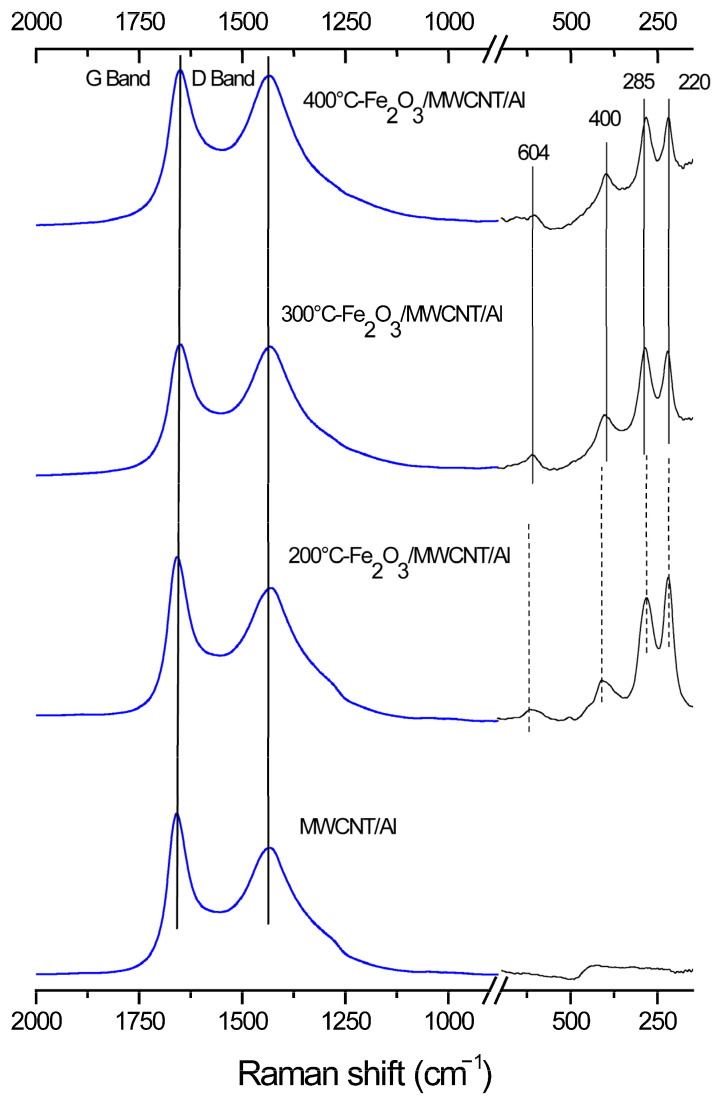
Raman shifts of MWCNT/Al and Fe_2_O_3_/MWCNT/Al (200, 300, and 400 °C).

The chemical structure of the composite material was studied using Raman spectroscopy and X-ray photoelectron spectroscopy. Figure 2 shows the Raman spectra for the MWCNT/Al (1) and Fe_2_O_3_/MWCNT/Al (2–4) composite material samples.

All spectra show two broad peaks at about 1335 and 1600 cm^−1^ [55], which are attributed to MWCNTs (D and G, respectively). Band G corresponds to the vibrations of the hexagonal crystal lattice of carbon, and band D characterizes the disorder of the MWCNT structure [56]. Since hematite belongs to the crystal space group D6 3d, the phonon modes A1g (220 cm^−1^) and Eg (285, 400 and 604 cm^−1^) can be distinguished in the spectra obtained [57]. The results show that three samples are covered with a layer of hematite. However, for the composite obtained at 200 °C, the decrease in the intensity of peak Eg1 (400 cm^−1^) to the intensity of peak Eg (285 cm^−1^), as well as a slight shift in peaks A1g and Eg (285 cm^−1^) towards the low-frequency region, may indicate the less perfect crystalline structure of the material [58]. This may also be indicated by a change in the ratio of the intensities of peaks A1g and Eg (285 cm^−1^) [59].

**Figure 3:**
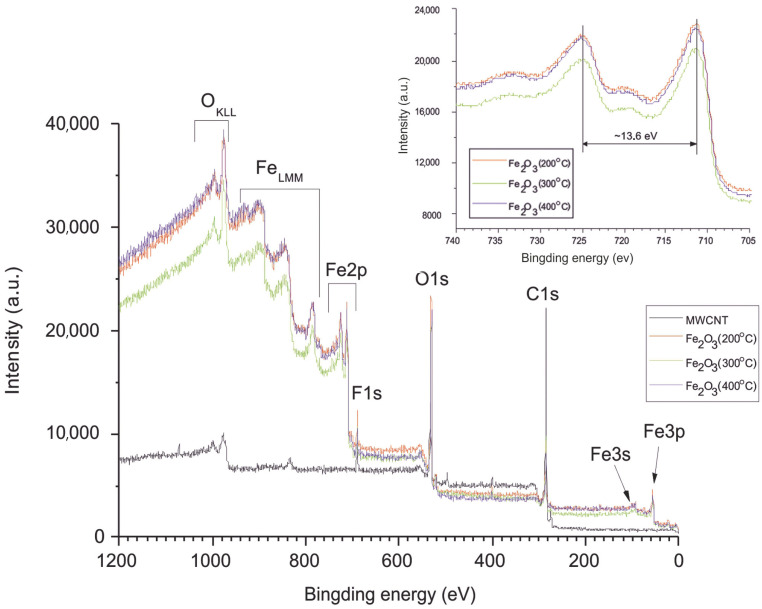
Long scan of the XPS spectrum with the inset showing the high-resolution Fe2p region of MWCNT/Al and Fe_2_O_3_/MWCNT/Al (200, 300, and 400 °C).

Typical XPS spectra are shown in Figure 3. The main intense lines in the survey spectrum with AlKα excitation are the series of iron, carbon, and oxygen spectra.

**Figure 4:**
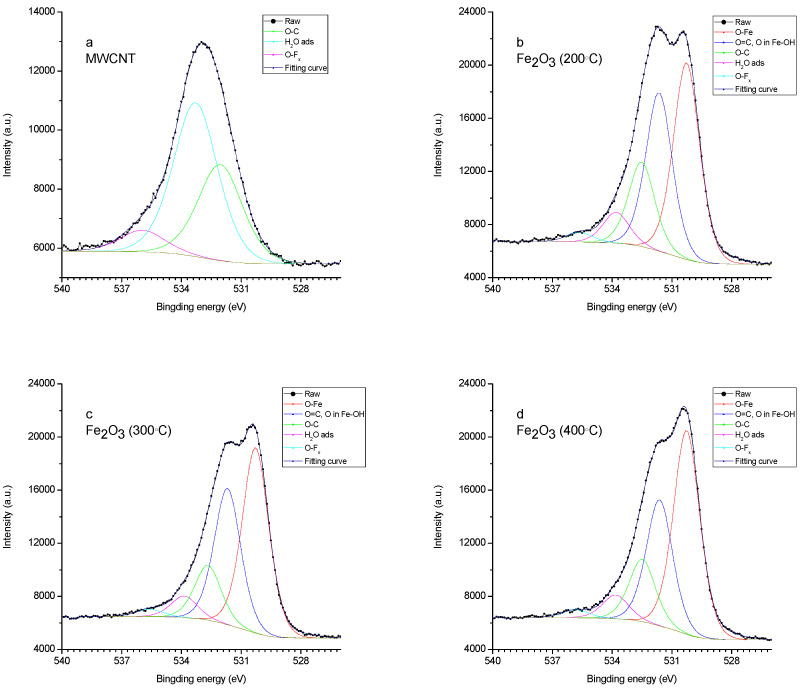
XPS spectrum (C1s) of (**a**) MWCNT/Al, (**b**) 200 °C—Fe_2_O_3_/MWCNT/Al, (**c**) 300 °C—Fe_2_O_3_/MWCNT/Al, and (**d**) 400 °C—Fe_2_O_3_/MWCNT/Al.

**Figure 5:**
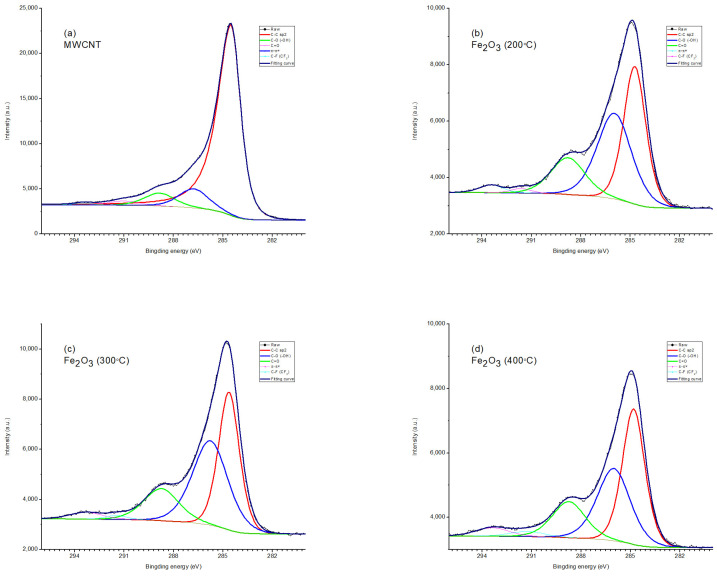
XPS spectrum (O1s) of (**a**) MWCNT/Al, (**b**) 200 °C—Fe_2_O_3_/MWCNT/Al, (**c**) 300 °C—Fe_2_O_3_/MWCNT/Al, and (**d**) 400 °C—Fe_2_O_3_/MWCNT/Al.

The high-resolution spectrum of Fe2p (Figure 3, inset) shows, for all iron-containing samples, two distinct peaks (Fe 2p 3/2 and 2p 1/2) separated by a broad satellite peak at 711.4 and 725 eV, respectively. The separation of the spin energies of Fe 2p 3/2 and 2p 1/2 is 13.6 eV, indicating that Fe in the composite is in the Fe^3+^ state [60]. Figure 4 and Figure 5 show the high-resolution XPS spectra of carbon (C1s) and oxygen (O1s). As can be seen from Figure 4, the spectra of carbon (C1s) are multi-component [61]. For sample 1, the spectrum corresponds to lightly oxidized carbon. The carbon in samples 2-4 is more oxidized. The C1s spectrum can be resolved into three peaks centered at 284.6 eV (C-C), 286.2 eV (C-O), and 288.6 eV (C=O) [62].

The oxygen spectrum is also multi-component (Figure 5). In sample 1, the main contribution comes from the bond lines with carbon and hydrogen in the water adsorbed by the sample. In samples 2-4, there are two lines (530.2 eV and 531.7 eV) associated with iron in the oxide and hydroxide [63]. In the sample obtained at an annealing temperature of 200 °C, the line corresponding to the hydroxide is more pronounced.

Based on the obtained data, it can be concluded that, with all methods of forming a composite material, a layer of Fe_2_O_3_ is formed on the surface of the carbon nanotube array. The optimal calcination stage is 300 °C.

### 3.2. Study of the Electrochemical Properties of Fe_2_O_3_/MWCNT/Al Composites

A series of electrochemical measurements were carried out to investigate the possibility of using the obtained Fe_2_O_3_/MWCNT/Al material as supercapacitor electrodes. The ability of a system to accumulate charge is usually determined by the specific capacitance (C_spm_) of the electrode materials and is calculated from data obtained with cyclic voltammetry methods [64]. A common unit of measurement for specific capacitance is F/g. The specific capacitance for the CV method is calculated using Equation (1).

C_spm_ = ∫ I d V: (ΔV × v × m_el_) (1)

In expression (1), ∫IdV is the total area under the CV curve, i.e., the accumulated charge; ΔV is the voltage range (V); ν is the scan rate (V/s); and m_el_ is the mass of the active material on the working surface of the electrode.

**Figure 6:**
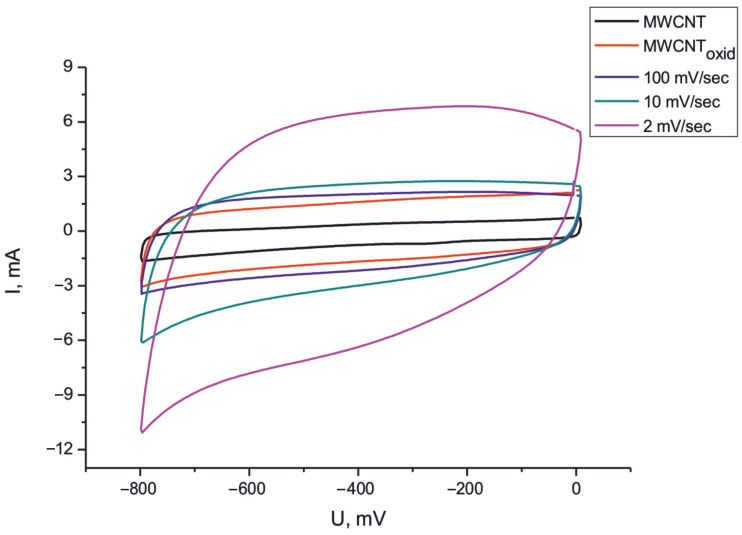
CVs of initial electrode (1), oxidized sample (2), Fe_2_O_3_/MWCNT/Al composite (100 mV/s) (3), Fe_2_O_3_/MWCNT/Al composite (10 mV/s), and (4) Fe_2_O_3_/MWCNT/Al composite (2 mV/s).

The cyclic voltammetry method showed a significant increase in the capacitance of the MWCNT/Al samples after the formation of the Fe_2_O_3_/MWCNT/Al composite material. Figure 6 shows examples of cyclic voltammograms for the initial MWCNT/Al samples, the electrochemically oxidized samples, and the composite material obtained at different voltage sweep rates.

From the plots shown in Figure 6, it is clear that the specific capacitance of the composite material Fe_2_O_3_/MWCNT/Al obtained at different voltage sweep rates (100, 10, and 2 mV/s) is significantly higher than that of the original MWCNT/Al sample. From the CV results, it can be concluded that the maximum increase in specific capacitance was achieved for the sample obtained at a voltage sweep rate of 2 mV/s (175 F/g at a scan rate of 100 mV/s and 230 F/g at a scan rate of 2 mV/s). No significant increase in specific capacitance was observed as the voltage sweep rate was increased. At 10 mV/s, the specific capacitance increased by a factor of about 3, and at 100 mV/s, it increased by a factor of about 2. The increase in the specific capacitance of the composite material is due to the pseudocapacitive properties of the iron oxide. Redox reactions take place at the electrode surface. Since an aqueous solution of sodium sulfate is used as the electrolyte, the charge storage mechanism can be described with the following equations [65,66,67,68]:

(Fe_2_O_3_)_surf_ + Na^+^ + e^−^ ‹–› (Fe_2_O_3_^−^, Na^+^)_surf_ (2)

(Fe_2_O_3_^−^, Na^+^)_surf_ + Na^+^ + e^−^ ‹–› (Fe_2_O_3_^2−^, 2Na^+^)_surf_ (3)

Fe_2_O_3_ + 2OH^−^ = Fe_2_O_3_ (OH)_2_ + 2e^−^ (4)

Fe_2_O_3_ + 2e^−^ + 3H_2_O = 2Fe (OH)_2_ + 2OH^−^ (5)

**Figure 7:**
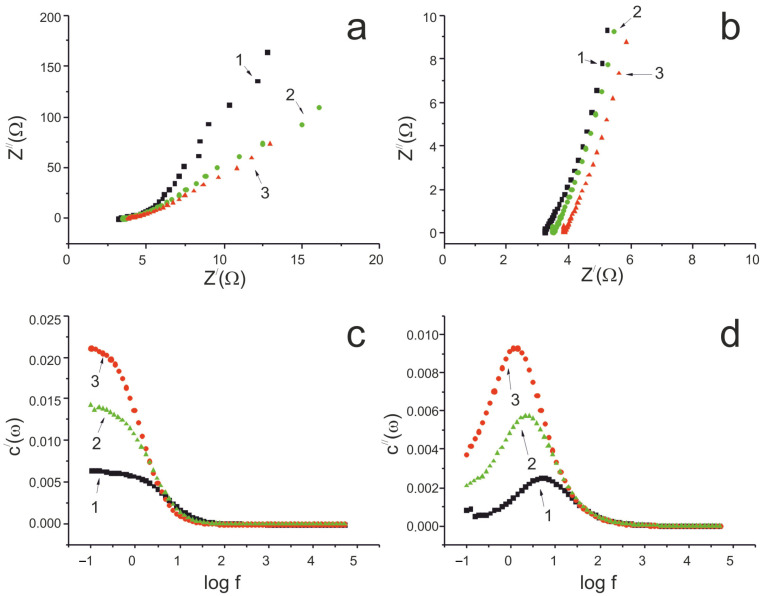
(**a**) Nyquist plots for a three-electrode cell (full range); (**b**) Nyquist plots for a three-electrode cell (middle range); (**c**) frequency dependence of real capacitance; and (**d**) frequency dependence of imaginary capacitance. As-prepared MWCNT/Al sample—1; oxidized MWCNT/Al sample—2; and Fe_2_O_3_/MWCNT/Al sample—3.

The electrochemical properties of the Fe_2_O_3_/MWCNT/Al electrodes were further investigated using electrochemical impedance spectroscopy. Figure 7a,b show Nyquist plots of the original MWCNT/Al sample, the pre-oxidized MWCNT/Al sample, and the Fe_2_O_3_/MWCNT/Al sample formed at a voltage sweep rate of 2 mV/s. The use of a Randles-like equivalent circuit is optimal for the obtained material [69,70,71].

In the low-frequency range, the Nyquist plots for the initial, oxidized, and composite Fe_2_O_3_/MWCNT/Al samples were almost linear, indicating good capacitive characteristics (Figure 7a). The ohmic resistance of the initial MWCNT/Al sample was 3.2 ohms, which increased to 3.5 ohms after electrochemical oxidation in a Na_2_SO_4_ solution. Further deposition of Fe_2_O_3_ on the MWCNT surface did not significantly increase the ohmic resistance (3.85 ohms). As a result, the Fe_2_O_3_ in the Fe_2_O_3_/MWCNT/Al composite electrode has good electrical contact with the conductive substrate. At the same time, the slope of the Nyquist plot for the Fe_2_O_3_/MWCNT/Al sample decreased in the low-frequency region compared to the original MWCNT/Al sample (Figure 7a), which can be explained by the pseudocapacitive properties of Fe_2_O_3_.

The frequency dependence of the actual capacitance confirms an increase in the electrode capacitance of about 1.5 times after electrochemical oxidation and about 5 times after Fe_2_O_3_ deposition (Figure 7c). This study showed that, during the anodic oxidation of MWCNTs, the increase in the specific capacity of the material is due to the increase in oxygen-containing groups covalently bonded to the surface of the nanotubes. Another reason is the increase in the porosity and specific surface area of the MWCNTs resulting from the electrochemical etching of the nanotube surface [52]. The specific capacitance of the Fe_2_O_3_/MWCNT/Al composite increases due to the oxidation reduction reactions taking place on the surface of electrodes (2) and (3). The imaginary capacitance dependence curves have maxima, the position of which characterizes the charge/discharge rate. The τ_r_ values for the initial MWCNT/Al electrode, the oxidized sample, and the final Fe_2_O_3_/MWCNT/Al sample were 0.22 s, 0.48 s, and 0.88 s, respectively. Pseudocapacitance limits rapid charge/ion transfer and results in a long response time. Therefore, the Fe_2_O_3_/MWCNT/Al electrode has a higher τ_r_ value of 0.88 s. However, when applied to pseudocapacitors, this value indicates the very good charge/discharge characteristics of the resulting binder-free composite electrodes [72].

**Figure 8:**
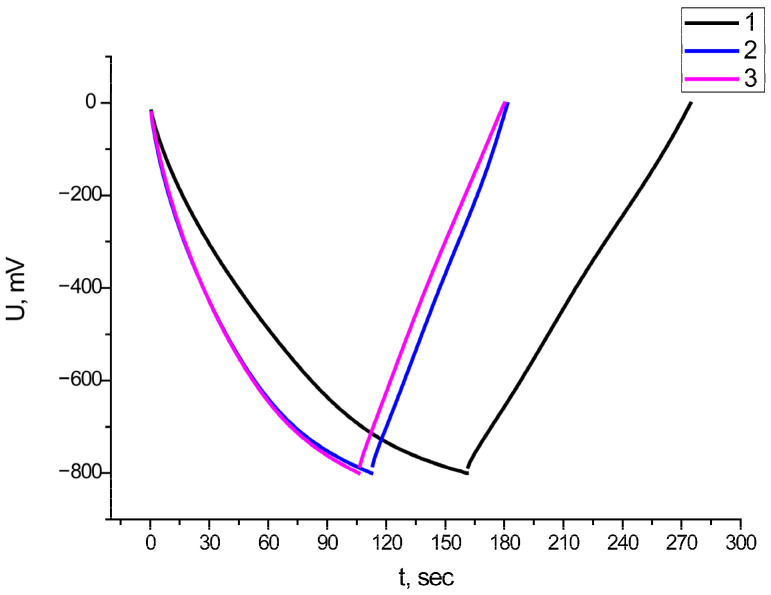
GCD of initial electrode: 1—composite obtained at a temperature of 200 °C; 2—composite obtained at a temperature of 300 °C; and 3—composite obtained at a temperature of 400 °C.

The galvanostatic charge/discharge (GCD) test is one of the most reliable methods for assessing specific capacity. Figure 8 shows the results of measuring the specific capacity for composite materials obtained at the calcination stages of 200, 300, and 400 °C. The specific capacity for this method is calculated using the following formula [64]:

C_spm_ = I/((dV/dt) m_el_) (6)

In expression (6), I is the applied current, dV/dt is the slope of the discharge curve, and m_el_ is the mass of the active material on the working surface of the electrode.

The average specific capacitance value for all composite materials was about 125 F/g at a current density of 2 A/g. The Coulomb efficiency was 70% for the composite material Fe_2_O_3_/MWCNT/Al obtained at the calcination stages of 200 and 400 °C, and it was 60% for the composite material Fe_2_O_3_/MWCNT/Al obtained at the calcination stage of 300 °C.

An important feature of SC electrodes is their resistance to multiple charge/discharge cycles. A series of measurements were taken at different calcination steps. Figure 8 shows the resistance of the Fe_2_O_3_/MWCNT/Al composite to multiple charge/discharge cycles as a function of annealing temperature.

**Figure 9:**
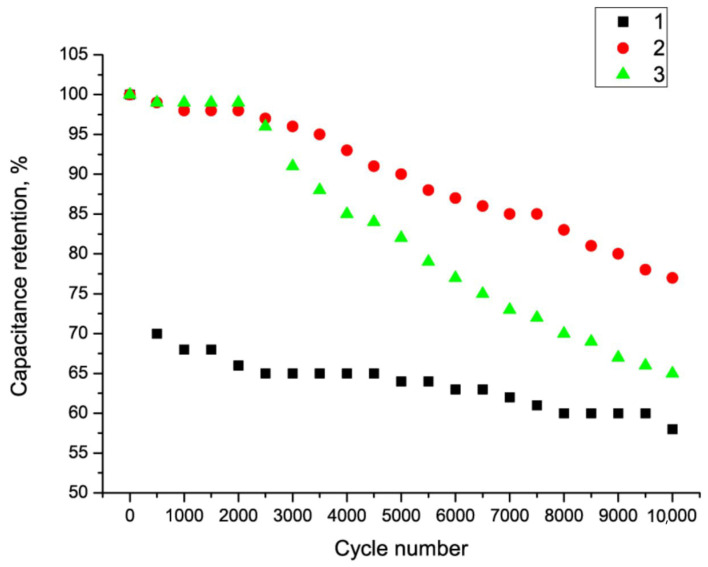
Capacitance evolution of Fe_2_O_3_/MWCNT/Al composite during multiple charge–discharge cycles as a function of annealing temperature: 1—composite obtained at a temperature of 200 °C; 2—composite obtained at a temperature of 300 °C; and 3—composite obtained at a temperature of 400 °C.

Figure 9 shows that the Fe_2_O_3_/MWCNT/Al composite exhibited the highest cycle resistance at an annealing temperature of 300 °C. These results suggest that annealing temperatures below 300 °C may not be sufficient to convert iron hydroxide to oxide. On the other hand, the MWCNT layer can be destroyed at a temperature of 400 °C.

As noted in the Introduction, many papers devoted to the study of supercapacitors made on the basis of carbon materials and iron oxide using various methods have been published. Therefore, it is interesting to compare the results of this work with previously published ones. Table 2 shows the literature data for various composite materials based on iron oxide.

It is evident that the values of the specific capacity of the composite materials we obtained are comparable to published values. At the same time, the composite material is highly resistant to numerous charge/discharge cycles. Undoubtedly, the main advantage of the method of direct formation of a composite material on a metal substrate is the possibility of obtaining a ready-to-use material for flexible electrodes of a supercapacitor.

## 4. Conclusions

In summary, a method for preparing a binder-free Fe_2_O_3_/MWCNT/Al composite electrode has been developed. For the first time, conditions for the electrochemical oxidation of MWCNTs grown directly on the surface of aluminum foil were selected to allow for the formation of an Fe_2_O_3_/MWCNT/Al composite. Fe_2_O_3_/MWCNT/Al electrodes without a binder were obtained via electrochemical oxidation of MWCNT/Al samples in a mixture of aqueous solutions (Fe(NH_4_)_2_(SO_4_)_2_ 0.1 M and CH_3_COONa 0.08 M). Electrochemical oxidation of MWCNT/Al samples in an aqueous solution (Fe(NH_4_)_2_(SO_4_)_2_ 0.1 M and CH_3_COONa 0.08 M) at a voltage sweep rate of 2 mV/s, followed by annealing at 300 °C, increases the specific capacitance of the active material of the samples by a factor of 5 to 175 F/g. At the same time, the studied material maintains excellent adhesion and electrical contact with the aluminum substrate. The Fe_2_O_3_/MWCNT/Al electrodes obtained have a high resistance to multiple charge/discharge cycles. After 10,000 cycles, the capacitance loss does not exceed 25%. The resulting material can be used as a ready-made flexible electrode (cathode) for a supercapacitor.

## Figures and Tables

**Table 1:** Concentrations of elements in the active layer of Fe_2_O_3_/MWCNT/Al samples depending on the voltage sweep rate.

Voltage Sweep Rate, mV/s	C, Mass.%	O, Mass.%	Fe, Mass.%
2	34.6	39.9	25.2
10	83.6	13.38	3.02
100	61.5	29.3	9.2

**Table 2:** Literary data. Parameter formation for composite material and corresponding electrochemical capacity values.

Substrate	Material Source; Deposition Method	Electrode Materials	Electrolyte Composition	CNT Specific Capacity, F/g	Cyclic Stability	Ref.
-	Fe(CO)_5_; evaporation	70% active materials, 20% carbon black, and 10% polyvinylidene fluoride	1 M Na_2_SO_4_	185	3000 (90.1%)	[17]
-	FeCl_3_·6H_2_O; hydrothermal synthesis	α-Fe_2_O_3_, HPS	1 M Na_2_SO_4_	465	4000 (88.4%)	[27]
Ni foam	solvothermal	a-Fe_2_O_3_, PPy	PVA/Na_2_SO_4_	1050 mF/cm^2^	10,000 (87.5%)	[29]
carbon fabric (CF)	solvothermal	Fe_2_O_3_-CF-6	1 M Na_2_SO_4_, PVA/LiCl	119	5000 (82.3%)	[14]
rGO-coated fabric	Fe(NO_3_)_3_, hydrothermal synthesis	Fe_2_O_3_/rGO	2 M KOH	360	8500 (89%)	[67]
Ni foam	Fe(NO_3_)_3__9H_2_O, solvothermal	Fe_2_O_3_/rGO	2 M KOH	1090 (195 for electrode)	5000 (67.3%)	[28]
Carbon Cloth (CC)	carbon cloth (CC), Ferric acetylacetonate, ethanol; flame synthesis	Ti-Fe_2_O_3_-CNT	1 M Na_2_SO_4_	1.25 F/cm^2^	3000	[48]
Al	(Fe(NH_4_)_2_(SO_4_)_2_ 0.1 M and CH_3_COONa 0.08 M) electrochemical deposition	Fe_2_O_3_/MWCNT/Al	0.5 M Na_2_SO_4_	175	10,000 (75%)	This work
